# Rule-based clinical decision support system for automated assessment of left ventricular diastolic function during stress echocardiography

**DOI:** 10.3389/frhs.2026.1690832

**Published:** 2026-04-15

**Authors:** Gulnora Rozikhodjaeva, Omonulla Juraev, H.-Christian Brauweiler, Tom Schaal

**Affiliations:** 1 Central Clinical Hospital No. 1 of the Main Medical Department Under the Administration of the Republic of Uzbekistan, Tashkent, Uzbekistan; 2 Center for the Development of Professional Qualifications of Medical Workers, Tashkent, Uzbekistan; 3 Faculty of Business and Economics, University of Applied Sciences Zwickau, Zwickau, Germany; 4 Faculty of Health and Healthcare Sciences, University of Applied Sciences Zwickau, Zwickau, Germany

**Keywords:** artificial intelligence, clinical decision support system, diastolic dysfunction, explainable AI, HFPEF, rule-based system, stress echocardiography

## Abstract

**Background:**

Heart failure with preserved ejection fraction (HFpEF) remains challenging to diagnose due to the complexity of diastolic function assessment during stress echocardiography, where multiple hemodynamic parameters must be evaluated under time pressure. Explainable artificial intelligence, specifically rule-based Clinical Decision Support Systems (CDSS), offers promising improvements in reproducibility and interpretability.

**Methods:**

A rule-based CDSS was developed and clinically validated to automate left ventricular diastolic function assessment during semi-supine bicycle stress echocardiography. A prospective cohort of 134 patients (mean age 61.3 ± 8.7 years) with exertional dyspnea and preserved left ventricular ejection fraction (LVEF >50%) was enrolled, excluding individuals with significant valvular pathologies, arrhythmias, or unstable ischemia. Echocardiographic and Doppler data were collected using Toshiba Aplio500 and Esaote MyLabSIGMA systems. The algorithm incorporated manual input of measurements, computed derived indices (e.g., diastolic reserve index, myocardial stiffness, vascular resistance), and applied rule-based logic in accordance with ASE/EACVI (2016/2022) guidelines and the ESC HFpEF consensus.

**Results:**

The CDSS generated diagnostic conclusions within 3 min per case, matching expert assessments in 93% of cases and correctly identifying stress-induced diastolic dysfunction in 85%. It demonstrated high diagnostic agreement (ICC > 0.94) and discrimination (AUC = 0.92). Rule-based outputs, such as “Impaired diastolic reserve” or “Right ventricular dysfunction under load,” were based on combinations of parameters (e.g., E/e′ > 15, Δe′ ≤ 0, TAPSE < 17 mm, PCWR > 12 mmHg).

**Conclusion:**

The explainable, guideline-compliant CDSS enables real-time, transparent analysis of diastolic function, supporting improved diagnostic consistency and augmented physician decision-making in cardiovascular care.

## 1 Introduction

Diastolic dysfunction of the left ventricle (LV) is a key diagnostic criterion for heart failure with preserved ejection fraction (HFpEF). The complex interaction of multiple echocardiographic parameters during stress echocardiography, coupled with strict time constraints, makes accurate interpretation challenging and prone to inter-observer variability (1, 2). Diastolic stress testing has been proposed to unmask dynamic abnormalities that are not evident at rest, providing critical information for the diagnosis of HFpEF (3). However, this method requires real-time acquisition, rapid calculations, and the integration of multiple indices, including transmitral flow, mitral annular velocities, pulmonary pressures, and cardiac output (1, 4).

In clinical practice, this process is often hindered by time constraints, complexity, and a lack of standardized decision-making tools. The interpretation of diastolic function under stress is especially vulnerable to operator dependency, and the accuracy of diagnosis may be reduced outside of expert centers. Artificial intelligence (AI) technologies offer new opportunities for automation of echocardiographic analyses. Although many approaches utilize deep learning (DL) algorithms, the lack of transparency and explainability (“black-box problem”) limits their adoption in clinical routines (5, 6). Alternatively, rule-based or symbolic AI methods enable knowledge-based reasoning and transparent logic flows, which are essential for clinical decision-making (7–11).

Explainable AI (XAI) approaches are increasingly viewed as essential for implementing trustworthy systems in medicine, especially in cardiology and imaging (12, 13). These tools can integrate domain knowledge and provide human-readable explanations for every diagnostic step, thereby supporting safety and regulatory requirements. Hence, the development of rule-based Clinical Decision Support Systems (CDSS) for stress echocardiography could address the key limitations of manual interpretation and opaque AI systems, enhancing accuracy, reproducibility, and clinical confidence (8, 14). The primary challenge addressed in this study is the high inter-observer variability and time-consuming nature of the diagnosis of HFpEF during stress echocardiography.

This study aimed to develop and validate a rule-based CDSS for automating the assessment of LV diastolic function during stress echocardiography and to compare its diagnostic performance with that of expert cardiologists.

## 2 Materials and methods

### 2.1 Study design and population

This prospective, single-center study was conducted between 2020 and 2022 at the Department of Functional Diagnostics, Central Clinical Hospital No. 1 of the Head Medical Department under the Administration of the President of the Republic of Uzbekistan. This study aimed to develop and validate a rule-based Clinical Decision Support System (CDSS) for the automated evaluation of left ventricular diastolic function during semi-supine bicycle stress echocardiography. A total of 134 adult patients (mean age 61.3 ± 8.7 years; 58.2% women) were consecutively enrolled based on clinical suspicion of HFpEF, specifically in the setting of exertional dyspnea with normal LVEF. Inclusion criteria were: age ≥ 18 years; preserved left ventricular ejection fraction (LVEF ≥ 50%) by biplane Simpson method; symptoms consistent with HFpEF: exertional dyspnea, fatigue, or exercise intolerance; sinus rhythm at rest; ability to complete exercise protocol.

The exclusion criteria were as follows: moderate to severe valvular heart disease, significant arrhythmias (e.g., atrial fibrillation/flutter), unstable coronary artery disease or recent myocardial infarction, acute or systemic inflammatory diseases, and poor echocardiographic imaging window.

All the participants provided written informed consent. The study was approved by the institutional ethics committee and conducted in accordance with the Declaration of Helsinki and Good Clinical Practice (GCP) guidelines.

### 2.2 Stress echocardiography protocol

All patients underwent semi-supine bicycle stress echocardiography with a standardized ramp protocol. The tests were performed under the supervision of experienced cardiologists and trained sonographers. The initial workload was set at 25 watts and increased in 25-watt increments every 3 min until symptom-limited maximum effort was achieved or clinical indications for termination were met. Echocardiographic data were acquired at baseline (resting conditions) and peak exercise. Imaging was performed using two commercial ultrasound systems: Aplio500 (Toshiba Medical Systems) and MyLab SIGMA (Esaote). The following echocardiographic modalities were used:

- Two-dimensional (2D) grayscale imaging
- Pulsed-wave Doppler for mitral inflow and LV outflow tract velocities
- Tissue Doppler Imaging (TDI) for mitral annular velocities (e′, a′, s′)
- Continuous-wave Doppler for tricuspid regurgitation velocity (TRV)
- Inferior vena cava (IVC) imaging for right atrial pressure estimation

All images and Doppler tracings were digitally recorded in the DICOM format and archived for subsequent expert review and processing using the CDSS.

### 2.3 Expert evaluation and reference standard

Two senior echocardiographers with over 10 years of clinical experience (high-level certification) independently analyzed all echocardiographic studies. They were blinded to each other's assessments and the CDSS outputs. Their individual interpretations were based on the current ASE/EACVI guidelines and included the calculation of key indices, such as the E/e′ ratio, TAPSE, Δe′, VTI, PCWR, and SVR.

Disagreements were resolved by consensus after a joint data review. The final expert consensus interpretation served as the reference standard for evaluating the diagnostic performance of the CDSS. All assessments were performed using vendor-independent, offline analysis software to minimize system variability.

### 2.4 Architecture of the rule-based CDSS

**Figure 1:**
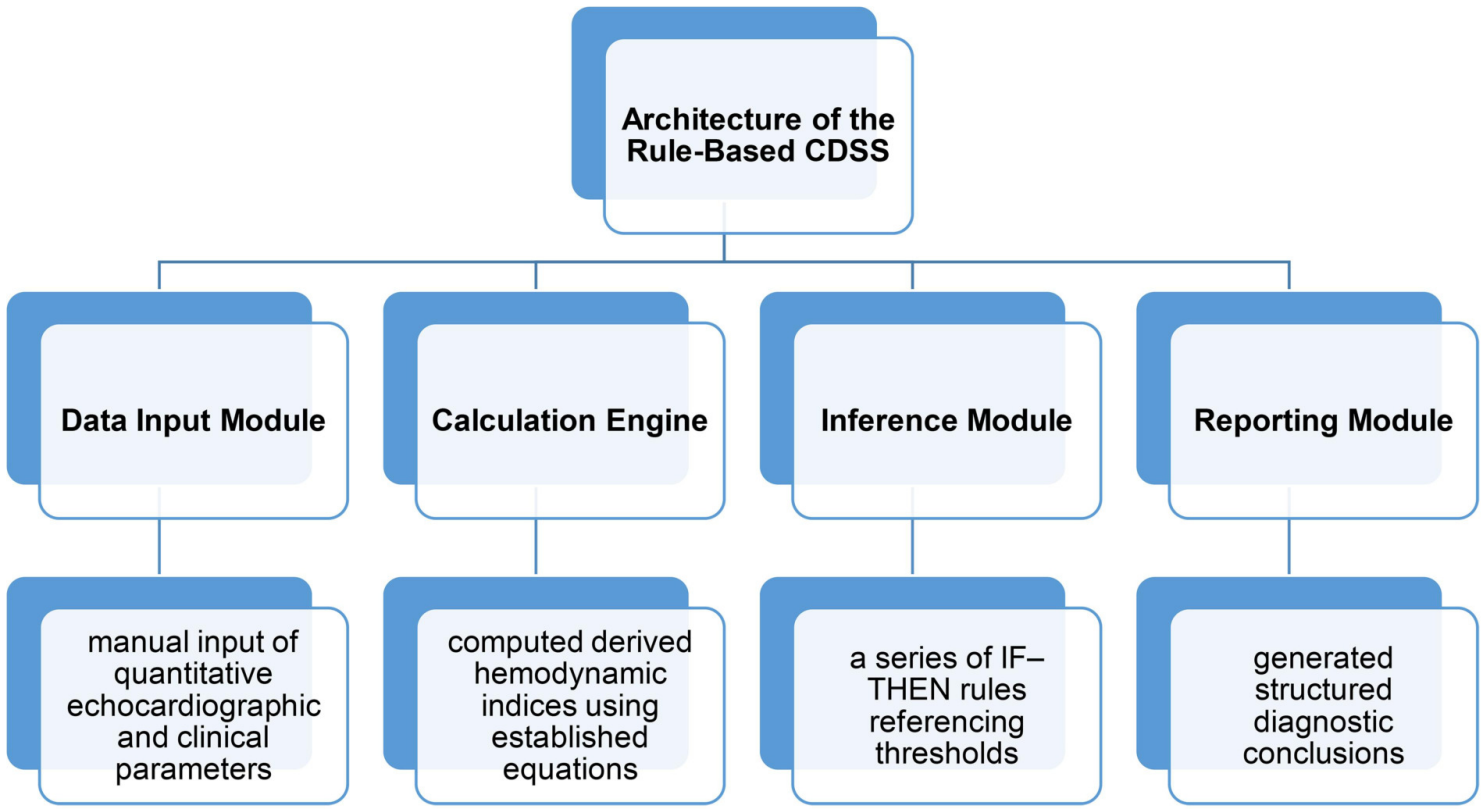
Architecture of the rule-based CDSS.

A Clinical Decision Support System (CDSS) was developed using a symbolic artificial intelligence (AI) approach based on deterministic rules derived from established echocardiographic guidelines. The system was implemented as a modular desktop application with four core components (Figure 1):

#### 2.4.1 Data input module

A structured interface for the manual input of quantitative echocardiographic and clinical parameters, including E/e′, e′ velocities, TAPSE, VTI, heart rate, blood pressure, and anthropometric data. The module includes validation checks for range limits and logical consistency.

#### 2.4.2 Calculation engine

This component computes the derived hemodynamic indices using established equations. Examples include:

Strokevolume(SV)=LVOTarea×VTI.

Cardiacoutput(CO)=SV×HR.

Cardiacindex(CI)=CO/BSA.

$$\mathrm{Pulmonary}\;\mathrm{capillary}\;\mathrm{wedge}\;\mathrm{resistance}\;(\mathrm{PCWR})\;=\;(E/e^{\prime })/\mathrm{SV}.$$

Systemicvascularresistance(SVR)=(MAP−RAP)/CO×80.

#### 2.4.3 Inference module

The core decision-making logic was implemented as a series of IF-THEN rules referencing thresholds from the ASE/EACVI and ESC HFpEF recommendations. For example:

IF Δe′ ≤ 0 AND E/e′ > 15 → THEN “Impaired diastolic reserve”.
IF PCWR> 12mmHg AND VTI<18 cm→THEN “Elevated afterload with low stroke volume” (Table 1).

**Table 1:** Summary of IF-THEN rules referencing thresholds.

Clinical rule	→	CDSS output
IF Δe′ ≤ 0 AND E/e′ > 15	THEN	Impaired diastolic reserve
IF TAPSE < 17 mm AND PCWR > 12 mmHg	THEN	Right ventricular dysfunction under load
IF SVR > 1,200 AND VTI < 18 cm	THEN	Reduced stroke volume with vasoconstriction
IF E/e′ ≤ 10 AND Δe′ > 1.5	THEN	Normal diastolic adaptation to stress
IF E/e′ > 15 AND TRV > 2.8 m/s	THEN	Elevated LV filling pressure
IF PCWR > 12 mmHg AND CO < 4.0 L/min	THEN	Post-load elevation with low output

The system integrates these metrics into a diagnostic framework that generates interpretable conclusions. Each conclusion was based on a combination of rules activated by specific parameter thresholds. This allows the CDSS to mimic clinical reasoning while providing fully traceable decision logic.

#### 2.4.4 Reporting module

This module generates structured diagnostic conclusions. It included traceable logic paths showing which rules were activated and flagged all the abnormal parameters. Each report included a summary section with clinical interpretations and reference guidelines.

The CDSS was run as a standalone executable program on Windows-based systems and was tested in an offline environment during clinical validation to ensure reproducibility and independence from network- or cloud-based services.

### 2.5 Parameters and thresholds used

**Figure 2:**
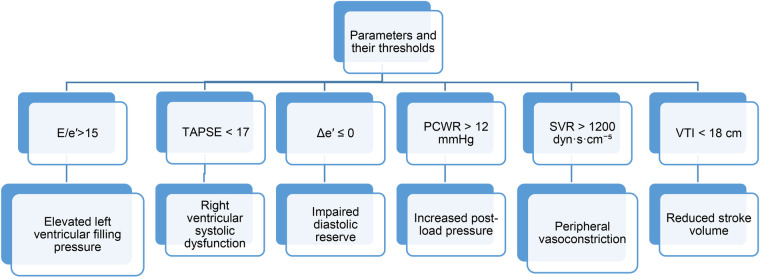
Parameters and thresholds were used for the classification.

The CDSS computes a comprehensive set of echocardiographic and hemodynamic parameters to evaluate diastolic function during stress. These include both directly measured and derived indices. The following parameters and thresholds were used for classification (Figure 2):

### 2.6 Statistical analysis

Statistical analyses were conducted using IBM Statistics SPSS (version 25.0, IBM Corp., Armonk, NY, USA) and MedCalc (version 20.0). Continuous variables are reported as mean ± standard deviation (SD), and categorical variables are expressed as absolute numbers and percentages. Agreement between the CDSS and expert consensus interpretation was evaluated using the interclass correlation coefficient (ICC) for continuous variables (e.g., E/e′, TAPSE, and PCWR) and Cohen's kappa coefficient (*κ*) for categorical outcomes (e.g., presence or absence of diastolic dysfunction). An ICC value >0.85 was considered indicative of excellent agreement. The diagnostic performance metrics included sensitivity, specificity, positive predictive value (PPV), negative predictive value (NPV), and area under the receiver operating characteristic curve (AUC) for the system's classification of probable HFpEF (2, 3).

The processing time (in minutes) from data input to report generation was recorded for each patient in the study. Statistical significance was set at *p* < 0.05.

## 3 Results

### 3.1 Patient characteristics

A total of 134 patients (mean age, 61.3 ± 8.7 years; range, 42–77 years) were included in the final analysis of this study. The clinical and demographic characteristics are summarized in Table 2.

**Table 2:** The clinical and demographic characteristics.

Parameter	Value
Total patients	134
Female sex (%)	86.2%
Mean BMI (kg/m^2^)	28.7 ± 3.2
Hypertension (%)	87.1%
Type 2 diabetes mellitus (%)	19%
Preserved LVEF (≥50%) (%)	100%
Exertional dyspnea (%)	91.0%
History of CAD (%)	27.6%
NYHA functional class I–II (%)	83.6%

### 3.2 CDSS output and decision profiles

**Figure 3:**
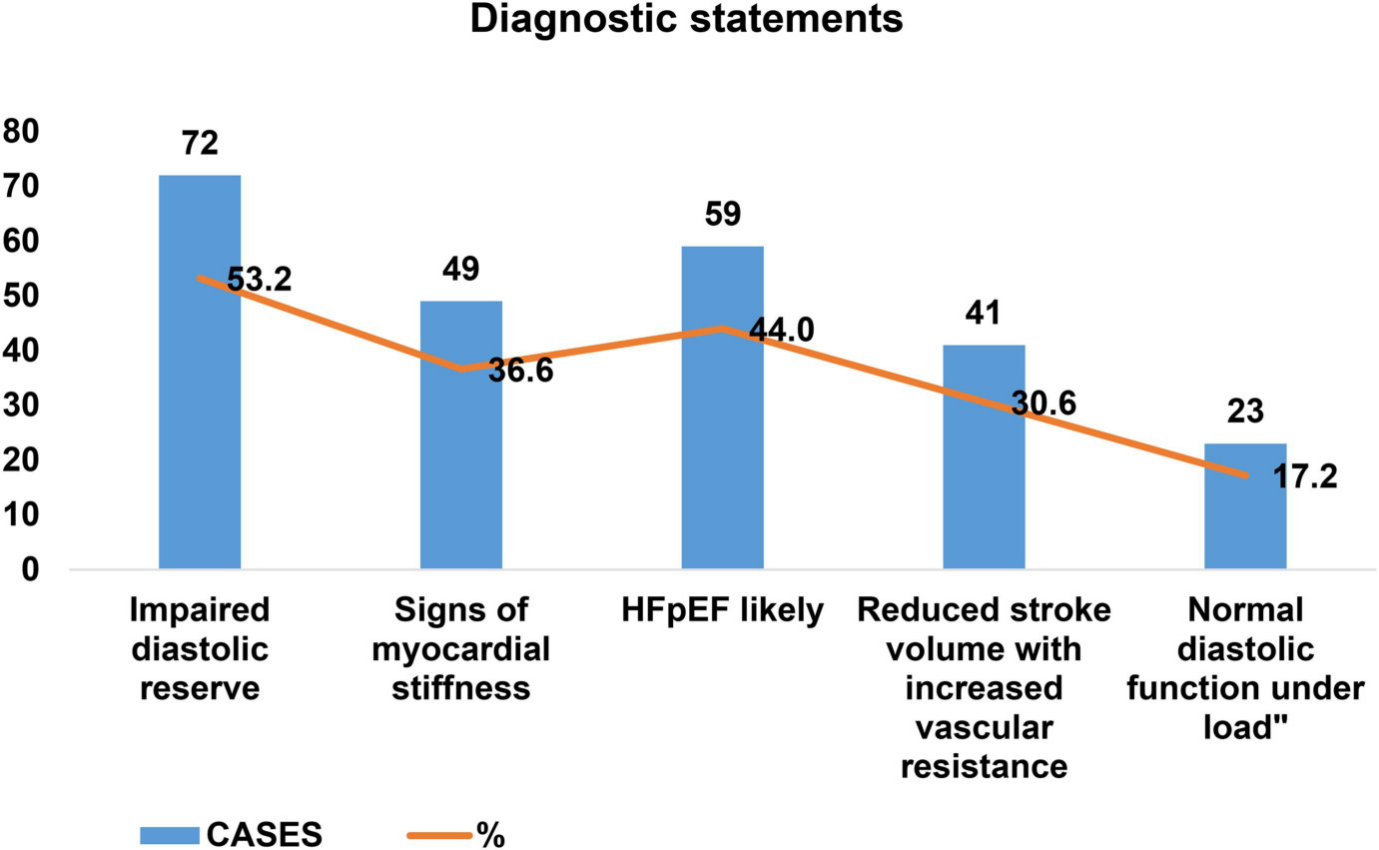
Frequency of different diagnostic patterns (cases-percentages).

The rule-based CDSS successfully generated diagnostic conclusions for all 134 cases without computational errors or interruptions. Based on predefined logic rules, the system provided structured reports with one or more of the following diagnostic statements: “Impaired diastolic reserve”—72 cases (53.7%); “Signs of myocardial stiffness”—49 cases (36.6%); “HFpEF likely”—59 cases (44.0%); “Reduced stroke volume with increased vascular resistance”—41 cases (30.6%); “Normal diastolic function under load”—23 cases (17.2%) (Figure 3).

Each conclusion included a rule trace summary with links to the criteria met, supporting explainability and auditability (9, 14).

### 3.3 Comparison with expert assessments

**Figure 4:**
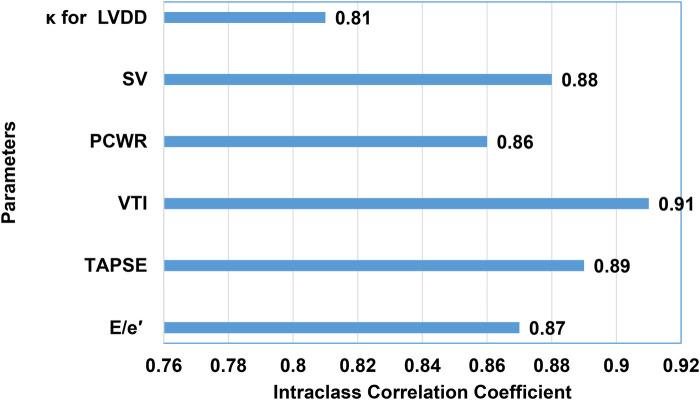
Agreement of results to expert assessment.

The results of the system were compared with the consensus diagnosis of two independent expert echocardiographers (Figure 4).

**Figure 5:**
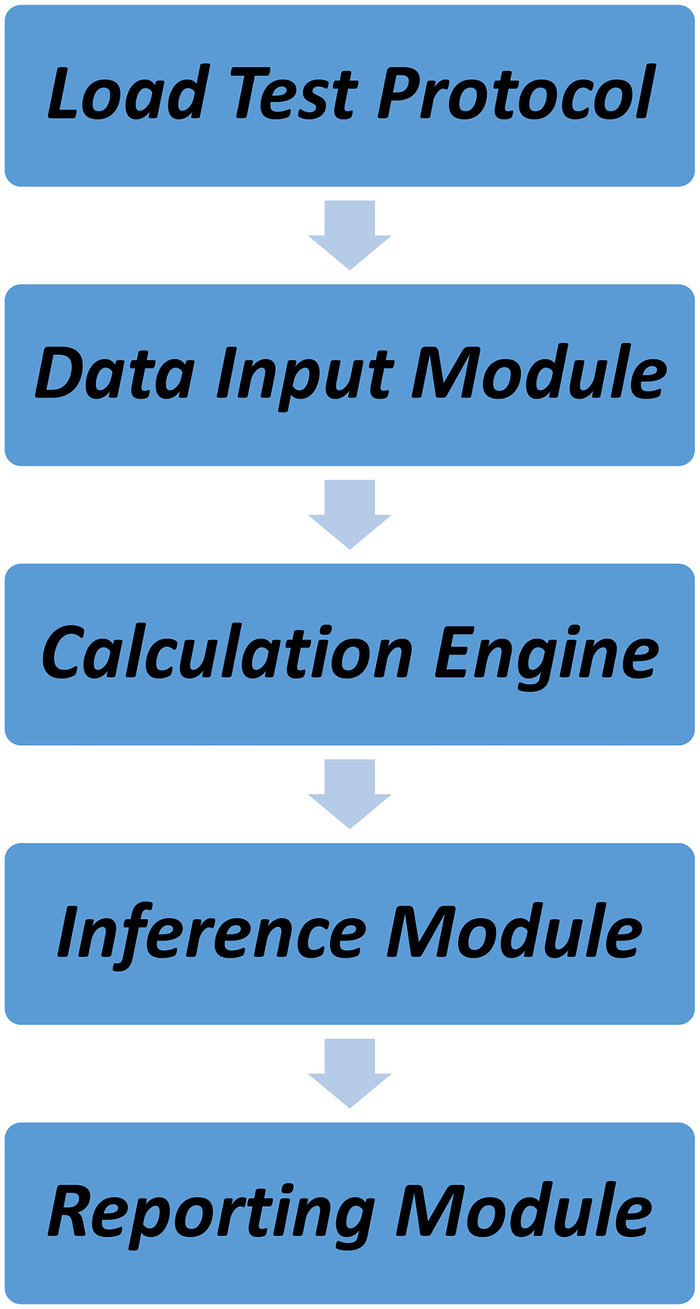
Overall design of the CDSS. Modules of the CDSS.

The diagram shows excellent agreement between the results obtained and the expert assessment (Figure 5).

#### 3.3.1 Agreement analysis

Interclass Correlation Coefficient (ICC):

E/e′: 0.87.
TAPSE: 0.89.
VTI: 0.91.
PCWR: 0.86.
SV: 0.88.

Cohen's Kappa (*κ*) for categorical classification of diastolic dysfunction: 0.81 (strong agreement).

#### 3.3.2 Overall match of the system vs. expert's final conclusion

The concordance rate was 93%. Discordant in 9 cases, of which 6 were borderline by expert judgment (e.g., Δe′ = 0.1).

### 3.4 Diagnostic performance

The CDSS performance for identifying diastolic dysfunction and probable HFpEF during stress echocardiography was evaluated as follows:

These metrics are comparable to previously published data for expert interpretation and surpass the performance of earlier AI-assisted tools in diastolic function analysis (4, 7).

### 3.5 Processing time and operational metrics

Average time from input to report generation: 2 min 46 s (166 s) per case (SD: ±31 s). This represents a >60% reduction in time compared to manual processing by experts (average of 7 min per case). System uptime: 100%. No runtime errors occurred during batch analysis.

### 3.6 Usability and explainability

A brief user survey (*n* = 5 cardiologists) was conducted to assess the practical usability and interpretability of the CDSS in a clinical setting.

- All users found the system's conclusions to be understandable and clinically relevant.
- Four out of five reported that the rule-tracing feature improved their confidence in borderline or ambiguous cases.
- All participants (*n* = 5) indicated that the interface was intuitive and could be integrated into the daily workflow without disruption.

All participants expressed interest in using the system in outpatient and functional testing settings.

The explainability of the CDSS was consistently rated as a strength. Users emphasized the value of transparent decision logic, especially in contexts where guideline adherence and reproducibility are critical (9, 14–17).

### 3.7 Illustrative case examples

To demonstrate how the CDSS operates across its functional modules in real clinical scenarios, two representative cases are presented (Tables 3–12). Each case illustrates the sequential activation of system modules from stress protocol initiation to final diagnostic synthesis.

**Table 3:** The CDSS performance for identifying diastolic dysfunction.

Metric	Value
Sensitivity	91.5%
Specificity	88.1%
Positive predictive value (PPV)	89.6%
Negative predictive value (NPV)	90.3%
Area under curve (AUC)	0.93 (95% CI: 0.89–0.97)

**Table 4.** Hemodynamic parameters and exercise test characteristics (Case A).

Date and time	Patient name	Birth year	Age	Weight (kg)	Height (cm)	BMI (kg/m^2^)	BSA (m^2^)
03/06/2026, 04:01:21	B.T.	1980	46	75	175	24.49	1.90

Parameter	Value	Hemodynamics	Rest	Peak	Dynamics (%)
Max. load	175 W	Heart rate, bpm	65	135	107.7
Stage, min	3	Systolic blood pressure, mmHg	120	155	29.2
Test duration	20:00	Diastolic blood pressure, mmHg	75	80	6.7
Recovery, min	5	Mean arterial pressure, mmHg	90.0	105.0	16.7
Submax HR	155	Double product	78.0	209.3	168.3
Tolerance	High	Termination criteria	Reached target HR		
Threshold load, W	175	On ECG	No ischemic changes		
Functional class	1	Reaction	Physiological		

**Table 5.** Echocardiographic parameters at rest and peak exercise (Case A).

Parameter	Rest	Peak	Dynamics (%)
Ascending aorta diameter (Ao), cm	3.1	3.2	3.2
Left atrial diameter (LA), cm	3.5	3.7	5.7
Indexed left atrial diameter (iLA), cm/m^2^	1.8	1.9	5.6
Interventricular septal thickness (IVS), cm	0.9	1.0	11.1
Posterior wall thickness (PW), cm	0.9	1.0	11.1
Left ventricular internal diameter in diastole (LVIDd), cm	4.8	5.0	4.2
Left ventricular internal diameter in systole (LVIDs), cm	3.1	2.9	−6.5
Left ventricular end-diastolic volume (LVEDV), mL	105	120	14.3
Indexed end-diastolic volume (EDVi), mL/m^2^	55.3	63.2	14.3
Left ventricular end-systolic volume (LVESV), mL	40	35	−12.5
Indexed end-systolic volume (LVESVi), mL/m^2^	21.1	18.4	−12.8
Right ventricular diameter (RV), cm	2.8	2.9	3.6
Ejection fraction (EF), %	62	71	14.5
Shortening fraction (SF), %	35	42	20.0
Stroke volume (SV), mL	72.7	90.1	23.9
Indexed stroke volume (iSV), mL/m^2^	38.3	47.4	23.8
Left ventricular myocardial mass (LVMM), g	140	145	3.6
Indexed left ventricular myocardial mass (iLVMM), g/m^2^	73.7	76.3	3.5
Relative wall thickness (RWT)	0.38	0.40	5.3
Left ventricular outflow tract diameter (LVOT), cm	2.1	2.1	0.0
Velocity-time integral (VTI), cm	21	26	23.8
Early transmitral flow velocity (E), cm/s	72	96	33.3
Late transmitral flow velocity (A), cm/s	55	70	27.3
E/A ratio	1.3	1.4	7.7
Early diastolic mitral annular velocity (e′), cm/s	12	19	58.3
Late diastolic mitral annular velocity (a′), cm/s	10	14	40.0
E/e′ ratio	6.0	5.1	−15.0
Maximum inferior vena cava diameter (IVC max), mm	18	19	5.6
Minimum inferior vena cava diameter (IVC min), mm	8	9	12.5
Inferior vena cava collapsibility index (IVC CI), %	55	55	0.0
Cross-sectional area of LVOT (CSA LVOT), cm^2^	3.5	3.5	0.0
Tricuspid regurgitation velocity (TR), m/s	2.2	2.6	18.2
Tricuspid regurgitation pressure gradient (TRPG), mmHg	19.3	27.0	39.9
Mitral regurgitation velocity (MR), m/s	0.5	0.5	0.0
Mitral regurgitation pressure gradient (MRPG), mmHg	2.0	2.5	25.0

**Table 6.** Derived hemodynamic and functional indices (Case A).

Parameter	Rest	Peak	Dynamics (%)
Left atrial volume (LAV), mL	45	48	6.7
Indexed left atrial volume (LAVi), mL/m^2^	23.7	25.3	6.8
Cardiac output (CO), L/min	4.7	12.2	159.6
Cardiac index (CI), L/min/m^2^	2.5	6.4	156.0
Minute distance (MD), cm/min	1,365	3,510	157.1
Tricuspid annular plane systolic excursion (TAPSE), mm	22	28	27.3
Diastolic reserve index (DRi)		3.7	
DRi/HR		0.1	
Left ventricular stiffness index (LVSI)	0.2	0.2	0.0
Systemic vascular resistance (SVR), dyn·s·cm^−5^	1,522.9	690.9	−54.6
Pulmonary capillary wedge pressure (PCWP), mmHg	9.3	8.2	−11.8

**Table 7.** Rule-based diagnostic criteria and thresholds applied by the CDSS (Case A).

Parameter	Rest	Peak	Parameter	Rest	Peak
E/e′ > 15	No	No	E/e′ > 15 + Δe′ = 0 + TR > 3 m/s	No	No
TAPSE < 17 mm	No	No	LAVi > 34 mL/m^2^	No	No
VTI < 18 cm	No	No	SVR > 1,200 + E/e′ > 15	No	No
VTI < 22 cm	No	No	Elevated resting SVR with normal CO and EF	No	No
PCWP > 12 mmHg	No	No			

**Table 8.** Hemodynamic parameters and exercise test characteristics (Case B).

Date and time	Patient name	Birth year	Age	Weight (kg)	Height (cm)	BMI (kg/m^2^)	BSA (m^2^)
03.06.2026, 03:23:21	XQ	1980	45	60	160	23.44	1.62

Parameter	Value	Hemodynamics	Rest	Peak	Dynamics (%)
Max. load	150 W (6-stage)	Heart rate, bpm	60	140	133.3
Stage, min	3	Systolic blood pressure, mmHg	110	180	63.6
Test duration	18:07	Diastolic blood pressure, mmHg	70	110	57.1
Recovery, min	6	Mean arterial pressure, mmHg	83.3	133.3	60.0
Submax HR	149	Double product	66.0	252.0	281.8
Tolerance	High	Termination criteria	Patient fatigue		
Threshold load, W	150	On ECG	No changes		
FC	1	Reaction	Physiological		

**Table 9.** Echocardiographic parameters at rest and peak exercise (Case B).

Parameter	Rest	Peak	Dynamics (%)
Ascending aorta diameter (Ao), cm	2.9	3.0	3.4
Left atrial diameter (LA), cm	3.4	3.8	11.8
Indexed left atrial diameter (iLA), cm/m^2^	2.1	2.3	9.5
Interventricular septal thickness (IVS), cm	1.0	1.1	10.0
Posterior wall thickness (PW), cm	1.0	1.1	10.0
Left ventricular internal diameter in diastole (LVIDd), cm	4.1	4.5	9.8
Left ventricular internal diameter in systole (LVIDs), cm	2.5	2.6	4.0
Left ventricular end-diastolic volume (LVEDV), mL	74.2	92.4	24.5
Indexed end-diastolic volume (EDVi), mL/m^2^	45.8	57.0	24.5
Left ventricular end-systolic volume (LVESV), mL	22.3	24.6	10.3
Indexed end-systolic volume (LVESVi), mL/m^2^	13.8	15.2	10.1
Right ventricular diameter (RV), cm	2.9	3.1	6.9
Ejection fraction (EF), %	69.9	73.4	5.0
Shortening fraction (SF), %	39	42.2	8.2
Stroke volume (SV), mL	90.5	100.9	11.5
Indexed stroke volume (iSV), mL/m^2^	55.9	62.3	11.4
Left ventricular myocardial mass (LVMM), g	132.1	175.0	32.5
Indexed left ventricular myocardial mass (iLVMM), g/m^2^	81.5	108.0	32.5
Relative wall thickness (RWT)	0.5	0.5	0.0
Left ventricular outflow tract diameter (LVOT), cm	2.4	2.6	8.3
Velocity-time integral (VTI), cm	20	19	−5.0
Early transmitral flow velocity (E), cm/s	92	121	31.5
Late transmitral flow velocity (A), cm/s	60	50	−16.7
E/A ratio	1.5	2.4	60.0
Early diastolic mitral annular velocity (e′), cm/s	6	6	0.0
Late diastolic mitral annular velocity (a′), cm/s	22	18	−18.2
E/e′ ratio	15.3	20.2	32.0
Maximum inferior vena cava diameter (IVC max), mm	30	36	20.0
Minimum inferior vena cava diameter (IVC min), mm	15	18	20.0
Inferior vena cava collapsibility index (IVC CI), %	50	50	0.0
Cross-sectional area of LVOT (CSA LVOT), cm^2^	4.5	5.3	17.8
Tricuspid regurgitation velocity (TR), m/s	3.1	3.5	12.9
Tricuspid regurgitation pressure gradient (TRPG), mmHg	38.4	49.0	27.6
Mitral regurgitation velocity (MR), m/s	0.95	0.9	−5.3
Mitral regurgitation pressure gradient (MRPG), mmHg	3.6	3.1	−13.9

**Table 10.** Derived hemodynamic and functional indices (Case B).

Parameter	Rest	Peak	Dynamics (%)
Left atrial volume (LAV), mL	34	36	5.9
Indexed left atrial volume (LAVi), mL/m^2^	21.0	22.2	5.7
Cardiac output (CO), L/min	5.4	14.1	161.1
Cardiac index (CI), L/min/m^2^	3.4	8.7	155.9
Minute distance (MD), cm/min	1,200	2,660	121.7
Tricuspid annular plane systolic excursion (TAPSE), mm	16	15	−6.3
Diastolic reserve index (DRi)		3.7	
DRi/HR		0.1	
Left ventricular stiffness index (LVSI)	0.21	0.22	4.8
Systemic vascular resistance (SVR), dyn·s·cm^−5^	1,228.0	755.3	−38.5
Pulmonary capillary wedge pressure (PCWP), mmHg	20.9	26.9	28.7

**Table 11.** Rule-based diagnostic criteria and thresholds applied by the CDSS (Case B).

Parameter	Rest	Peak	Parameter	Rest	Peak
E/e′ > 15	Yes	Yes	E/e′ > 15 + Δe′ = 0 + TR > 3 m/s	Yes	Yes
TAPSE < 17 mm	Yes	Yes	LAVi > 34 mL/m^2^	No	No
VTI < 18 cm	No	No	SVR > 1,200 + E/e′ > 15	Yes	No
VTI < 22 cm	Yes	Yes	Elevated filling pressure despite preserved CO and EF	Yes	Yes
PCWP > 12 mmHg	Yes	Yes			

**Table 12:** Agreement between CDSS outputs and expert cardiologist assessments.

Parameter	Expert assessment	CDSS assessment	Agreement (ICC)
E/e′ ratio	Elevated filling pressure	Elevated filling pressure	0.88
TAPSE	RV function preserved/reduced	Same classification	0.86
VTI	Normal/reduced stroke volume	Same classification	0.87
PCWR	Elevated/normal	Same classification	0.89
Overall diastolic function classification	Expert consensus	CDSS output	0.85

Two illustrative cases were selected to demonstrate the decision pathway of the proposed CDSS under physiological and pathological hemodynamic conditions.

#### 3.7.1 Case A—physiological adaptation to load

##### 3.7.1.1 Load test protocol module

Semi-supine bicycle ergometry was performed using a standard protocol. The test was terminated due to patient fatigue. No electrocardiographic abnormalities were observed.

##### 3.7.1.2 Data input module

The echocardiographic and clinical parameters were entered.

##### 3.7.1.3 Calculation engine

##### 3.7.1.4 Inference module

No pathological rule clusters were activated.

The system identified preserved diastolic reserve and physiological vascular adaptation.

##### 3.7.1.5 Reporting module

The CDSS automatically generated the following structured clinical interpretation:

- Normal diastolic adaptation to exercise. No evidence of HFpEF.
- Normal left ventricular filling pressure at rest and during exercise.
- Preserved diastolic reserve.
- Normal myocardial stiffness.

#### 3.7.2 Case B—HFpEF with impaired reserve

##### 3.7.2.1 Load test protocol module

The stress protocol was completed under similar standardized conditions.

##### 3.7.2.2 Data input module

##### 3.7.2.3 Calculation engine

##### 3.7.2.4 Inference module

##### 3.7.2.5 Reporting module

The CDSS produced the structured clinical conclusion:

- Hemodynamic profile is consistent with HFpEF criteria.
- Stress-induced elevation of left ventricular filling pressure.
- Diastolic reserve, including indexed reserve, is impaired.
- Impaired left ventricular filling due to increased myocardial stiffness.

The proposed rule-based CDSS correctly differentiated physiological adaptation to exercise (Case A) from HFpEF with impaired diastolic reserve (Case B).

## 4 Discussion

The development and implementation of a rule-based Clinical Decision Support System (CDSS) for the automated assessment of left ventricular diastolic function during stress echocardiography represents a clinically interpretable and guideline-aligned approach to addressing diagnostic complexity in patients with preserved ejection fraction. In the present study, the CDSS demonstrated high diagnostic agreement with expert cardiologists (ICC > 0.85) and strong discriminative performance (AUC = 0.93), supporting its value as a structured decision-support tool in real-world cardiology practice (4, 6).

The evaluation of diastolic function, particularly under dynamic physiological conditions such as exercise stress, requires the integration of multiple interdependent parameters, including E/e′ ratio, tissue Doppler velocities, TAPSE, VTI, pulmonary capillary wedge pressure (PCWR), and vascular resistance indices (1, 2, 12, 18). Although international recommendations provide structured diagnostic algorithms (1, 12), their application in stress conditions remains complex and operator-dependent. Manual interpretation in time-constrained environments, such as functional diagnostic laboratories, is therefore vulnerable to inter-observer variability and diagnostic inconsistency. The CDSS presented in this study addresses these limitations by automating parameter calculation, standardizing threshold-based logic, and generating structured, reproducible conclusions aligned with ASE/EACVI and ESC HFpEF consensus documents (1, 12, 19).

In recent years, artificial intelligence has significantly advanced echocardiographic analysis, particularly through deep learning-based image interpretation and automated functional quantification (17, 19, 25–27). However, despite impressive performance metrics, many neural network-based systems lack interpretability and provide limited transparency regarding the reasoning underlying their predictions. In high-stakes domains such as cardiology, this opacity may reduce clinician trust and complicate regulatory approval (10, 14). In contrast, the present CDSS adopts a deterministic rule-based architecture, ensuring that each diagnostic conclusion is directly traceable to predefined guideline-derived thresholds.

This approach aligns with contemporary principles of trustworthy artificial intelligence, which emphasize transparency, auditability, human oversight, and risk management in clinical AI systems (2). Unlike black-box models operating on latent image features, the proposed system processes structured numerical inputs and applies explicit decision rules, thereby allowing clinicians to verify each step of the inference process. For example, the conclusion “impaired diastolic reserve” results from the logical co-occurrence of Δe′ ≤ 0, elevated PCWR, and reduced VTI-each parameter independently measurable and clinically interpretable. This traceable reasoning pathway enhances reproducibility and supports clinician confidence.

Importantly, rule-based clinical decision support systems remain highly relevant in structured diagnostic environments where adherence to established guidelines is essential (11). While deep learning approaches may excel in pattern recognition tasks, deterministic symbolic architectures offer advantages in interpretability, portability, and regulatory transparency. The modular design of the CDSS-comprising data acquisition, derived hemodynamic computation, rule activation and clustering, and structured reporting-further strengthens interpretability and facilitates integration into existing clinical workflows.

Time efficiency represents another clinically meaningful advantage. The average automated calculation and report generation time was approximately 3 min, rendering the system feasible for high-throughput outpatient and functional diagnostic settings. In busy clinical environments, standardized computational support may reduce cognitive load and improve consistency without replacing expert clinical judgment.

Receiver operating characteristic (ROC) analysis confirmed the robustness of the CDSS, demonstrating high sensitivity and specificity relative to expert consensus (AUC = 0.93), thereby supporting its role as a triage and validation instrument (7, 16, 20). Agreement metrics further indicate that structured rule-based automation can approximate expert-level interpretation while maintaining transparency.

An additional perspective that may further enhance cardiovascular risk stratification involves the consideration of thoracic morphological indices. In particular, *the* modified Haller index (MHI) has recently been proposed as a non-invasive anthropometric parameter reflecting chest wall conformation and anteroposterior thoracic diameter. Previous studies suggest that individuals with reduced thoracic depth may exhibit distinctive cardiopulmonary interactions that can influence cardiac chamber geometry and functional measurements during exercise. Assessment of MHI under stress conditions may therefore provide complementary insights into thoracic mechanics and cardiovascular performance during physical exertion. Incorporating structural thoracic parameters together with functional echocardiographic indices may represent a promising direction for future development of multimodal clinical decision support systems (21).

Several limitations of this study should be acknowledged. First, the current system relies on manual input of echocardiographic and clinical variables, which may introduce operator-dependent variability. Future integration with automated data extraction from echocardiographic workstations and PACS systems could improve workflow efficiency and reduce the potential for input-related inconsistencies.

Second, this investigation represents a single-center validation study conducted using two ultrasound platforms. Although the CDSS processes standardized echocardiographic variables rather than vendor-specific imaging data, broader validation across multiple institutions, patient populations, and ultrasound vendors is required to confirm the robustness and generalizability of the system.

Third, while the rule-based architecture ensures transparency and interpretability, future hybrid models combining explainable rule-based logic with machine learning-assisted parameter extraction may further enhance diagnostic performance.

### 4.1 Generalizability and external validation

Although the present study was conducted in a single tertiary center using two ultrasound platforms, the CDSS architecture was intentionally designed to be vendor-independent. The system does not rely on raw image data, proprietary software features, or device-specific post-processing algorithms. Instead, it operates on standardized numerical echocardiographic parameters (e.g., E/e′, tissue Doppler velocities, TAPSE, VTI, heart rate, blood pressure) that are universally available across contemporary echocardiographic systems and defined within international recommendations (7, 12, 22–24).

Because the inference engine applies deterministic threshold-based logic derived from ASE/EACVI and ESC HFpEF guidelines, its functionality is not restricted to a specific manufacturer or institutional workflow. This structured approach enhances portability and reduces the risk of model performance degradation due to cross-vendor variability, a limitation frequently observed in deep learning-based imaging systems (17, 19, 20).

Nevertheless, external validation across multiple centers, devices, and patient populations remains essential. Differences in acquisition protocols, operator expertise, and demographic characteristics may influence measured parameters and downstream inference. Prospective multicenter studies incorporating diverse datasets will therefore be required to confirm robustness, reproducibility, and clinical scalability of the proposed CDSS. Importantly, the rule-based architecture facilitates recalibration if population-specific adjustments become necessary, thereby supporting adaptive deployment without compromising transparency.

## 5 Conclusion

In conclusion, the proposed rule-based CDSS represents a transparent, guideline-aligned, and clinically interpretable form of artificial intelligence in cardiology. By combining deterministic reasoning, structured reporting, and high diagnostic agreement with expert assessment, the system offers a safe and reproducible approach to stress-induced diastolic function evaluation. This study demonstrates that a rule-based Clinical Decision Support System (CDSS) can deliver a fast, reliable, and explainable analysis of diastolic function during stress echocardiography.

The system achieved excellent agreement with expert interpretations, reduced diagnostic time by over 60%, and strictly adhered to the international guidelines. Its architecture aligns with the principles of trustworthy AI, including transparency, safety, and human-centered design.

Although not intended to replace clinicians, the CDSS offers a reproducible and structured diagnostic framework that supports decision-making in real-world cardiovascular practice. It bridges the gap between manual assessment and fully automated black-box models, providing a safe and interpretable AI solution for echocardiographic diagnoses.

## Data Availability

The raw data supporting the conclusions of this article will be made available by the authors, without undue reservation.
